# Mosaicism of Podocyte Involvement Is Related to Podocyte Injury in Females with Fabry Disease

**DOI:** 10.1371/journal.pone.0112188

**Published:** 2014-11-11

**Authors:** Michael Mauer, Emily Glynn, Einar Svarstad, Camilla Tøndel, Marie-Claire Gubler, Michael West, Alexey Sokolovskiy, Chester Whitley, Behzad Najafian

**Affiliations:** 1 Department of Pediatrics, University of Minnesota, Minneapolis, United States of America; 2 Department of Medicine, University of Minnesota, Minneapolis, United States of America; 3 Department of Pathology, University of Washington, Seattle, United States of America; 4 Department of Medicine, Haukeland University Hospital, Bergen, Norway; 5 Department of Clinical Medicine, University of Bergen, Bergen, Norway; 6 Department of Pediatrics, Haukeland University Hospital, Bergen, Norway; 7 U983, Université René Descartes, Hôpital Necker-Enfants Malades AP-HP, Paris, France; 8 Division of Nephrology, Department of Medicine, Dalhousie University, Halifax, Nova Scotia, Canada; University of Houston, United States of America

## Abstract

**Background:**

Fabry disease. an X-linked deficiency of α-galactosidase A coded by the GLA gene, leads to intracellular globotriaosylceramide (GL-3) accumulation. Although less common than in males, chronic kidney disease, occurs in ∼15% of females. Recent studies highlight the importance of podocyte injury in Fabry nephropathy development and progression. We hypothesized that the greater the % of podocytes with active wild-type GLA gene (due to X-inactivation of the mutant copy) the less is the overall podocyte injury.

**Methods:**

Kidney biopsies from 12 treatment-naive females with Fabry disease, ages 15 (8–63), median [range], years were studied by electron microscopy and compared with 4 treatment-naive male patients.

**Results:**

In females, 51 (13–100)% of podocytes (PC) per glomerulus had no GL-3 inclusions, this consistent with a non-Fabry podocyte phenotype (NFPC). In PC with GL-3 inclusions [Fabry podocyte phenotype (FPC)], GL-3 volume density per podocyte was virtually identical in females and males, consistent with little or no cross-correction between FPC and NFPC. %NFPC per glomerulus (%NFPC/glom) correlated with age in females (r = 0.65, p = 0.02), suggesting a survival disadvantage for FPC over time. Age-adjusted %NFPC/glom was inversely related to foot process width (FPW) (r = −0.75, p = 0.007), an indicator of PC injury. GL-3 volume density in FPC in females correlated directly with FPW.

**Conclusions:**

These findings support important relationships between podocyte mosaicism and podocyte injury in female Fabry patients. Kidney biopsy, by providing information about podocyte mosaicism, may help to stratify females with Fabry disease for kidney disease risk and to guide treatment decisions.

## Introduction

Fabry disease is a storage disease caused by deficiency of the α-galactosidase A (αGal A) enzyme that hydrolyzes the terminal α-galactosyl moieties from glycolipids and glycoproteins. This leads to the accumulation of its substrates, predominately globotriaosylceramide (GL-3) in various cell types and organs, causing a constellation of complications including skin lesions, strokes, cardiac arrhythmias and cardiomyopathy, neuropathies and renal failure. [1] αGal A is encoded by the GLA gene located on the X chromosome locus Xq21.3-q22. Similar to other X-linked diseases, the complications are typically less frequent and more variable in severity in females, [2], [3], [4] although they can be as severe as in male patients. [5], [6] A significant proportion of female patients suffer from important complications, including 40% with clinical renal disease (mainly proteinuria) [7] and about 15% with serious renal events. [2] Fabry disease is associated with significant life expectancy reductions in both sexes. [8] It is of great importance to understand the factors associated with disease severity in females. Currently, there are no reliable tests to identify females at greater risk to develop kidney failure, thus justifying earlier treatment with enzyme replacement therapy (ERT). Podocytes are terminally differentiated cells with pivotal role in preserving glomerular structure and function. [9] Recent studies suggest that GL-3 accumulation in podocytes plays an important role in the pathophysiology of Fabry nephropathy. [10] These cells are also much more resistant to ERT than most other kidney cell types. [11], [12] Similar to other “terminally differentiated cells” podocytes do not easily regenerate following injury [13]. Continuous podocyte loss leads to progressive reduction of these cells in the glomeruli, this eventually reaching critical levels causing irreversible glomerular scarring [14]. Despite recent evidence that higher doses of ERT during childhood may result in partial to almost complete clearance of podocytes from GL-3 inclusions, [15] there is no consensus as to when to initiate ERT, especially in females, and the relative clinical effectiveness of the different licensed ERT doses remain unsettled. [16], [17], [18] We hypothesized that podocytes, due to random X-inactivation, are heterogeneously involved by Fabry disease in female patients and that this heterogeneity could influence podocyte injury. Herein we describe a method to quantify the % of podocytes with the Fabry phenotype and report an inverse relationship in females between age-adjusted % podocytes with no GL-3 inclusions in glomeruli and foot process width, a sensitive indicator of podocyte injury [19], supporting a relationship between X-inactivation and podocyte injury in females with Fabry disease. We also found no evidence of cross-correction between podocytes without and with the Fabry phenotype in females with Fabry disease.

## Methods

These studies were performed in accordance with principles of the Declaration of Helsinki and were reviewed and approved by the Institutional Review Board of the University of Minnesota, Comité. de Protection des Personnes “Ile-De-France II.” and the Regional Ethics Committee of Western Norway. Informed consents approved by the institutional board review committees were obtained prior to these studies.

### Subjects and Clinical Parameters

Kidney biopsies from 12 ERT-naive females with Fabry disease, age 15 (8–63) years were studied by electron microscopy for distribution of podocyte involvement by the Fabry phenotype. Biopsies were obtained for assessment of the severity of the lesions of Fabry nephropathy in order to aid clinical decision-making regarding ERT initiation and/or as a baseline biopsy prior to ERT initiation. Biopsies from 4 ERT-naive males with Fabry disease, age 14 (7–18), were studied for comparison; 7/16 patients presented here were included in our previous publications. [10], [15] The demographic and clinical data of all patients are presented in Table 1.

**Table 1:** Clinical characteristics of subjects.

**Case**	**Sex**	**Age (year)**	**UPCR** **(mg/g)**	**UACR** **(mg/g)**	**GFR** **(ml/min/1.73 m^2^)**	**GLA** **Mutation**	**Mutation** **Type**	**Aangiokeratoma**	**Corneal** **Opacity**
1	F	8	40	NA	NA*	NA	NA	+	+
2	F	11	0.02	53	105	c.800T>G(p.M267R)	Missense	+	+
3	F	12	29	NA	109	W236X	Nonsense	−	−
4	F	13	0	NA	97	Y216D	Missense	−	+
5	F	13	60	5	100	NA	NA	+	−
6	F	14	62	11	90	c.800T>G(p.M267R)	Missense	NA	NA
7	F	16	30	12	127	NA	NA	−	−
8	F	34	150	NA	118	R301Q	Missense	+	−
9	F	34	100	5	99	R112C	Missense	−	+
10	F	39	100	NA	78	1270T	Missense	+	+
11	F	39	40	ND	99	N215S	Missense	+	−
12	F	63	1150	NA	46	C.427G>Cp.Ala143Pro	Missense	+	−
13	M	7	0	NA	183	Y216D	Missense	+	+
14	M	16	92	12	112	c.1212_1214delAAG	Deletion	+	+
15	M	18	251	135	96	c.800T>G(p.M267R)	Missense	+	+
16	M	23	102	NA	111	NA	NA	NA	NA

Abbreviations: UPCR = urine protein/creatinine ratio; UACR = urine albumin/creatinine ratio; GFR = glomerular filtration rate; NA = data not available; *Serum creatinine within the normal range; F = female; M = male; ND = Not detectable.

9/12 female and 3/4 male patients had results of GLA mutation analysis in their medical records confirming the diagnosis of Fabry disease (Table 1). The diagnosis of Fabry disease in the other patients was based on family history or clinical findings with or without reduced leukocyte αGal A activity and confirmed by the kidney biopsy findings. Protein excretion per gram creatinine (UPCR) was based on urine samples obtained close to the date of biopsy. GFR was estimated by the plasma clearance of iohexol where available or by creatinine clearance. Except for one of the patients where we were not able to find information about the use of renin-angiotensin system blockers, none of the patients were receiving these drugs.

Biopsies from 6 healthy living kidney donors, age 37 (16–52) were used to estimate normal control values for podocyte foot process with (FPW), as described below.

### Biopsy Tissue Preparation and Electron Microscopy

Electron microscopy specimens were fixed in 2.5% glutaraldehyde, and embedded in PolyBed. Random glomerular sections were prepared as previously described. [20] Thin sections were mounted on formvar coated copper slot grids. Overlapping digital low magnification (∼10,000 x) images of the entire glomerular profiles were obtained using a JEOL CX100 electron microscope for the podocyte mosaicism studies. High magnification (∼30,000 x) images were obtained according a systematic uniform random sampling protocol for estimation of fraction of the volume (Vv) of PC cytoplasm occupied by GL-3 inclusions [Vv(Inc/PC)], Vv of inclusions/glomerular mesangial cell [Vv(Inc/Mes)], Vv of inclusions/glomerular endothelial cell [Vv(Inc/Endo)], and podocyte average FPW as previously described [10], [20].

### Identification of Podocytes with and without the Fabry Phenotype

Observers were masked to any of the patient characteristics. Montages of complete glomerular profiles were prepared from the above images in Adobe Photoshop software (Adobe Photoshop CS5 Extended, version 12.0×32). Twice digital magnification was applied to the images. Podocyte nuclei were identified and glomerular profiles with less than 10 podocyte nuclei (n = 3) were excluded from these studies. The cytoplasmic profiles surrounding each podocyte nucleus were carefully examined for presence of GL-3 inclusions. Podocyte nuclear profiles with cytoplasmic GL-3 inclusions, consistent with Fabry phenotype podocytes (FPC) or without cytoplasmic GL-3 inclusions, consistent with non-Fabry phnotype podocytes (NFPC) cytoplasmic GL-3 inclusions were counted in each glomerulus and %NFPC/glom was calculated. The maximum number of immediately adjacent podocytes, including podocytes on the other side of the same capillary loop, which were NFPC was recorded as an estimate of the size of podocyte mosaic patches on the section.

### Electron Microscopy Stereology

**Figure 1:**
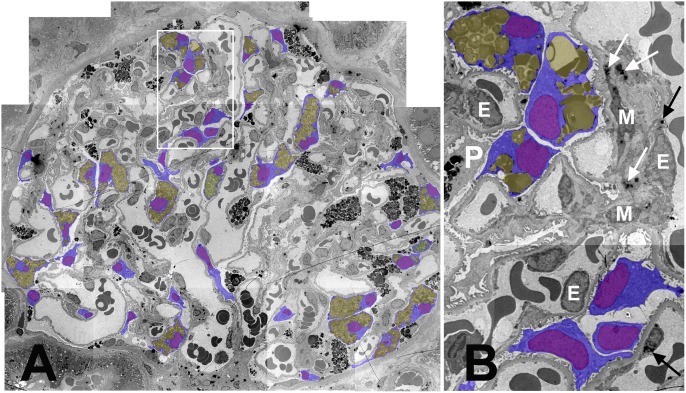
Mosaicism of podocyte Fabry phenotype in a glomerulus from a female patient with Fabry disease. (**A**) Montage image of a glomerulus (∼3,000×). Podocyte bodies with visible nuclei are colored blue, podocyte nuclei purple, and GL-3 inclusions yellow. The white rectangle is magnified in B. (**B**) Magnified view of three podocyte profiles without (at the bottom) and three other podocyte profiles with GL-3 inclusions (on the top). Arrows show GL-3 inclusions in mesangial (M) cells (black) and endothelial (E) cells. P is a podocyte profile with no visible nucleus on this section.

Based on the best quality of tissue preservation and images, one glomerulus per biopsy was arbitrarily selected for detailed GL-3 volume density measurement in podocytes with visible nuclei on the section. Boundaries of nuclei and cell membranes, excluding the tertiary foot processes were traced using the magnetic lasso tool in separate layers in Adobe Photoshop (Figure 1). Similarly, the most convex points of cytoplasmic GL-3 inclusions were connected using the magnetic lasso tool to draw a polygon around the GL-3 inclusion aggregates in a separate layer. The tracings were colored differentially for nuclei, cytoplasm and inclusions (Figure 1). The observed magnification was calculated from the average of 10 horizontal and 10 vertical random measurements performed on images from a SPI grating carbon replica #02902-AB (Structure Probe, Inc., West Chester, PA, USA) with horizontal and vertical lines 0.463 µm apart obtained at the same magnification as for the montage images. Subsequently, the measurement tool of the software was calibrated. The area of cell, nucleus and inclusion aggregate profiles were obtained separately for each podocyte profile with a visible nucleus from the Adobe Photoshop measurement log. The fractional volume of GL-3 inclusions per each nucleated podocyte profile with GL-3 inclusions [Vv(Inc/FPC)] was estimated as follows: 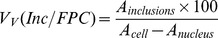. The overall average glomerular volume fraction of GL-3 inclusions per podocytes [Vv(Inc/PC)], endothelial cells [Vv(Inc/Endo)] and mesangial cells [Vv(Inc/Mes)] were estimated using unbiased stereology methods as previously detailed. [10] For clarity, we emphasize that Vv(Inc/FPC) is an estimate of GL-3 inclusion density in nucleated podocyte profiles with GL-3 inclusions, while Vv(Inc/PC) is an estimate of the same parameter in all visible podocyte profiles regardless of GL-3 content and including the podocytes with and without visible nuclei over 3 (1–3), median (range), glomeruli per biopsy. Average foot process width (FPW) was also estimated using unbiased stereology methods as detailed elsewhere. [10], [20], [21] In order to compare podocyte injury in FPC *vs.* NFPC in female patients with Fabry disease, FPW was separately estimated in systematically and uniformly obtained electron micrographs with and without FPC.

### Statistical Analyses

Statistica 8.0 (Statsoft, Inc.) was used for statistical analysis. Comparison between groups was made by student t-test after confirming homogeneity of variances. Relationships between variables were evaluated using Pearson correlation. Partial correlations were performed to control for confounding variables. Random effects model variance component analysis was performed to estimate % contribution of biopsies (inter-subject) and glomeruli (intra-subject) variations to total variance of %NFPC per glomerulus (%NFPC/glom). p<0.05 was considered statistically significant.

## Results

### Electron Microscopy Examination of Kidney Biopsies

Examination of electron micrographs from all biopsies allowed easy and reliable distinction between male and female patients by the identification in females of podocytes with visible nuclei without GL-3 cytoplasmic inclusions, termed non-Fabry podocytes (NFPC) (Figure 1) in contrast with podocytes containing GL-3 inclusions, termed Fabry phenotype podocytes (FPC). However, mosaicism of the Fabry phenotype was not easily identifiable in the other glomerular or extra-glomerular cells, perhaps, at least in part, due to uncertainty about cellular boundaries (*e.g*., mesangial or endothelial cells). In 4 female patient biopsies, no GL-3 inclusions were identified in endothelial and/or mesangial cells, thus, comments about mosaicism could not be made in those cell types. In 7/12 female patient biopsies, parietal epithelial cell profiles with no GL-3 inclusions were identified while occasional parietal epithelial cells with enlarged cytoplasm had abundant GL-3 inclusions. Characteristic lamellar GL-3 inclusions were not easily identified in proximal tubular epithelial cells. Distal tubular epithelial cells showed variable GL-3 inclusions in both males and females. Thus, this variability could not be accounted for X-inactivation mosaicism in those cells.

### Distribution of GL3 Inclusions among Podocytes in Males and Females

54 (27–87) podocytes per biopsy were examined for presence of GL-3 inclusions in female patients. 51 (13–100)% of podocyte profiles per biopsy with visible nuclei and no GL-3 inclusions, were classified as NFPC in these females. NFPC were distributed as single cells among FPC or in patches composed of 2–6 podocytes (Figure 1).

Two biopsies from female patients (one with 3 and another with 2 glomeruli) had no GL-3 inclusions in podocytes with visible nuclei (cases #9 and #11, Table 1). Case #9 showed rare podocyte profiles that were filled with GL-3. However, because these GL-3 containing podocytes had no visible nuclei they were not included in calculation of %NFPC per glomerulus. This case had a missense mutation (R112C) with GFR, UPCR and UACR values all within the normal range (Table 1). Clinical examination revealed corneal opacities, but no angiokeratoma. Echocardiograms showed normal left ventricular size and function with an estimated ejection fraction of 55–60% and normal right ventricular size and systolic function. Case #11 had distal tubular cells with GL-3 inclusions. This case had a cardiac variant mutation (N215S) with normal GFR and UPCR values and no detectable albumin in the urine (Table 1). The diagnosis of Fabry disease was made based on known family history. Clinical examination revealed angiokeratoma, but no corneal opacities. Clinically, she was asymptomatic. No echocardiograms were available from this case. None of these two subjects had a history of stroke. In order to determine whether random sectioning through podocytes may have obscured the observation of GL-3 inclusions in podocyte profiles 4 male patients with Fabry disease were similarly studied, 1–3 (median 2.5) glomeruli, containing 18–36 (median 22) podocyte profiles per glomerulus with visible nuclei were examined (Table 1). All podocyte profiles from these glomeruli contained abundant GL-3 inclusions, consistent with FPC, except for two very small profiles in a 7-year-old boy. The volume fraction of GL-3 inclusions per FPC [Vv(Inc/FPC)] was nearly identical in males (0.56±0.11) and females (0.53±0.13; p = 0.54).

### Inter- and Intra-subject Variations of Podocyte Phenotype Mosaicism

**Figure 2:**
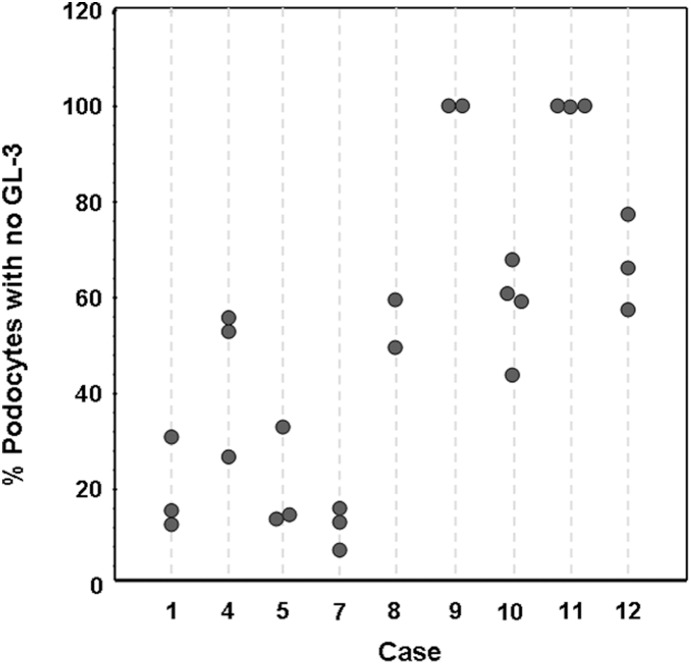
Intra- and inter-subject variability of podocyte mosaicism for Fabry phenotype in females. X axis shows case numbers (see Table 1). Each vertical dashed line represents a biopsy and each circle represents % podocytes with no GL-3 inclusions in one glomerulus.

Biopsies from 9 female patients with more than one glomerulus (3 (2–5), median (range)) available for electron microscopy were used to compare inter- and intra- subject variability of podocytes with the Fabry the phenotype. The %NFPC/glom in 2–4 glomeruli per biopsy in female patients is shown in Figure 2. Variance component analysis showed that only 9.6% of total variance in podocyte Fabry phenotype mosaicism originated from inter-glomerular (intra-subject) variation, while the vast majority of variance lay in differences in this parameter among the subjects.

### Relationships Between % Podocytes with no GL-3 Inclusions and Female Patient Characteristics, Renal Function and Other Glomerular Structural Parameters

**Figure 3:**
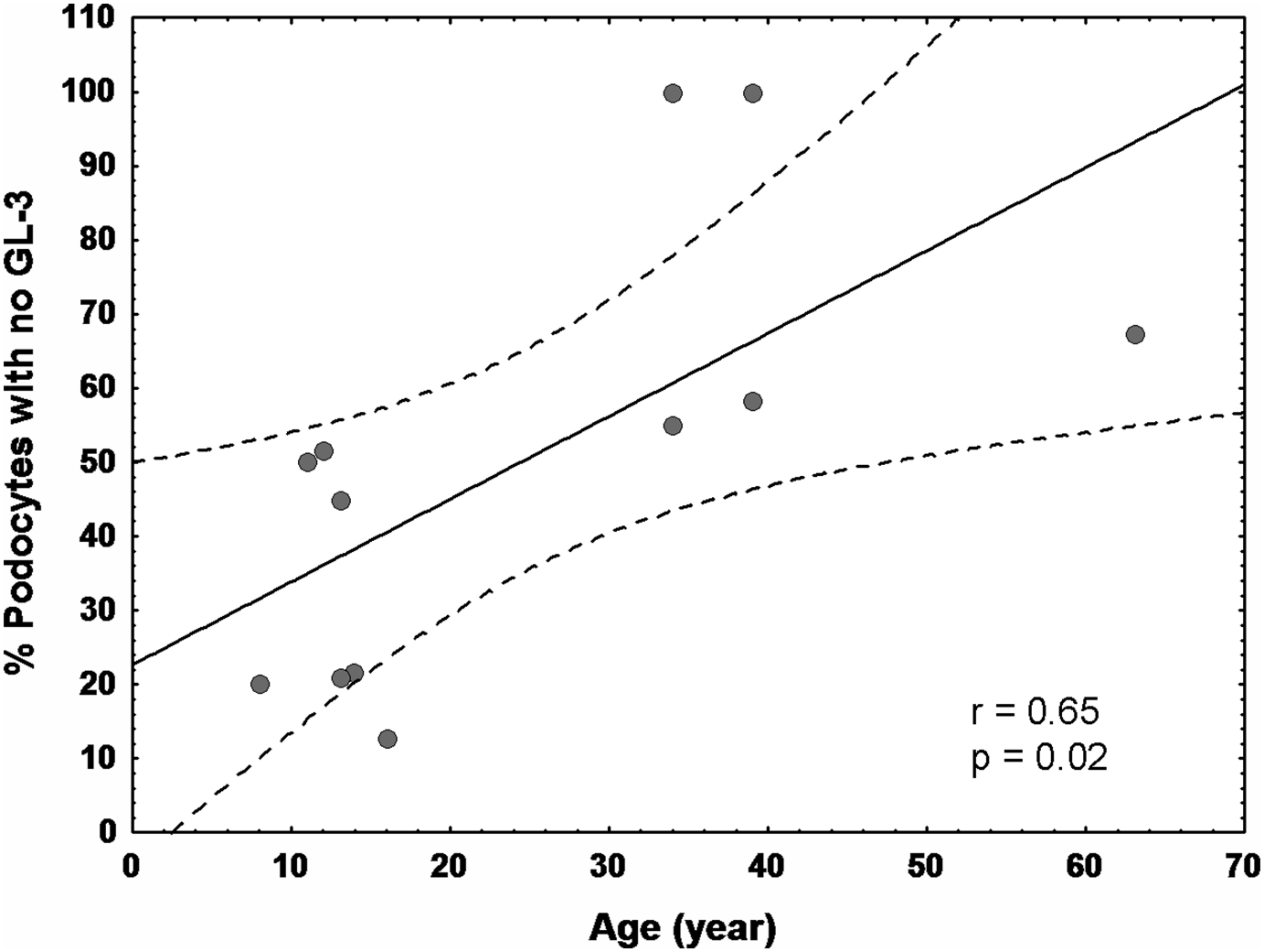
Relationship between age and % podocytes with no GL-3 in females. Dashed lines represent 0.95 confidence interval.

Values of %NFPC/glom, Vv(Inc/PC), volume fraction of GL-3 inclusions per mesangial cells [Vv(Inc/Mes)], volume fraction of inclusions per endothelial cells [Vv(Inc/Endo)], and foot process width (FPW) are provided in Table 2. The average %NFPC/glom was calculated in biopsies from female patients with more than one available glomerulus. There was a direct relationship between age and %NFPC/glom (r = 0.65; p = 0.02, Figure 3). No statistically significant relationship was found between %NFPC/glom and urine albumin/creatinine ratio (UACR) (available in 6/12 patients), urine protein creatinine ratio (UPCR) or glomerular filtration rate (GFR). As expected, %NFPC/glom was inversely related to Vv(Inc/PC) (r = −0.70, p = 0.02) for all podocytes. Simple linear regression analysis revealed no statistically significant relationships between %NFPC/glom and foot process width, Vv(Inc/Endo), Vv(Inc/Mes). However, adjusted for age, significant inverse relationships were found between %NFPC/glom and FPW (r = −0.75, p = 0.007), and Vv(Inc/Mes) (r = 0.70, p = 0.02), but not with Vv(Inc/Endo).

**Table 2:** Glomerular Structural Parameters.

Case	Sex	Age (year)	%NFPC/glom	Vv(Inc/PC)	Vv(Inc/Mes)	Vv(Inc/Endo)	Vv(Inc/FPC)	FPW* (nm)
1	F	8	20	0.28	0.03	0.08	0.50	524
2	F	11	50	0.43	0.02	0	0.62	426
3	F	12	52	0.21	0	0	0.42	372
4	F	13	45	0.20	0	0.03	0.42	464
5	F	13	21	0.26	0.03	0.03	0.68	663
6	F	14	22	0.38	0.07	0.02	0.53	602
7	F	16	13	0.51	0.03	0.02	0.67	461
8	F	34	55	0.43	0.01	0.01	0.70	620
9	F	34	100	0	0	0.01	0	427
10	F	39	58	0.21	0.01	0	0.35	654
11	F	39	100	0.02	0.01	0	0	441
12	F	63	68	0.16	0.04	0.06	0.48	673
13	M	7	8**	0.21	0.01	0.09	0.58	369
14	M	16	0	0.44	0.10	0.37	0.56	551
15	M	18	0	0.50	0.16	0.21	0.61	823
16	M	23	0	0.34	0.56	0.27	0.65	714

Abbreviations: %NFPC/glom = % non-Fabry podocytes per glomerulus; Vv(Inc/PC) = volume fraction of GL3 inclusions per podocyte; Vv(Inc/Mes) = volume fraction of GL3 inclusions per mesangial cell; Vv(Inc/Endo) = volume fraction of GL3 inclusions per endothelial cell; Vv(Inc/FPC) = volume fraction of GL3 inclusions per Fabry podocytes; FPW = foot process width; F = female; M = male; *Normal values for FPW obtained from biopsies from 6 healthy living donors was 406±34 nm. **The biopsy contained two very small podocyte profiles with no GL3 inclusions, most likely due to random sectioning.

**Figure 4:**
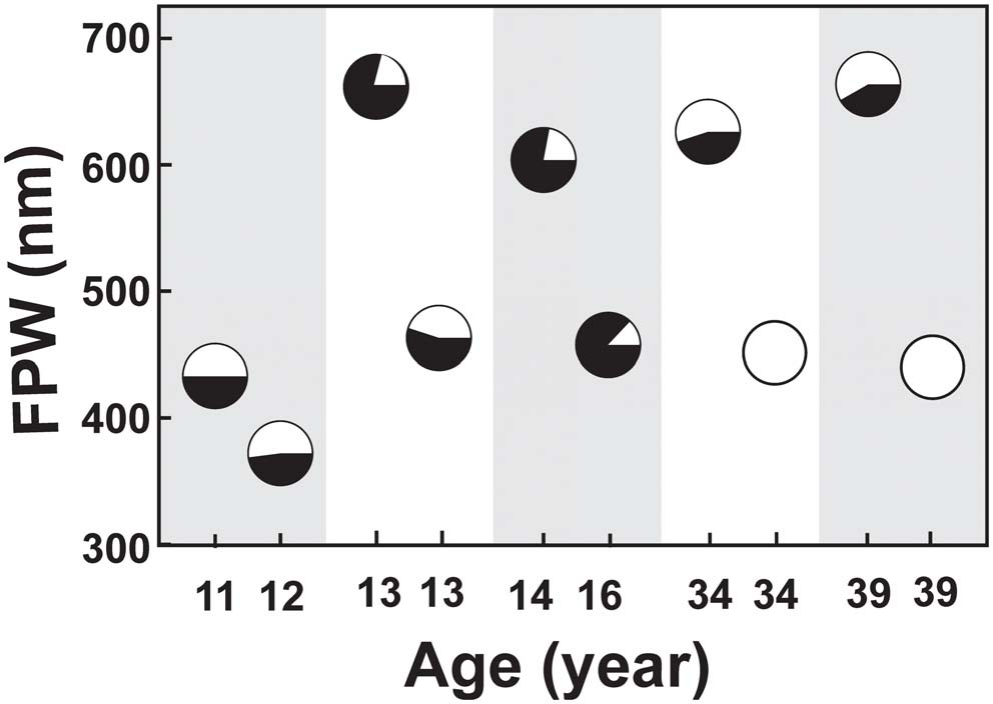
Relationships between podocyte mosaicism and foot process width in age-matched paired females. Each vertical grey or white band represents an age-matched pair. Each pie chart represents %NFPC/glom as white and %FPC/glom as black area. Abbreviation: FPW: foot process width.

To better visualize the relationships between %NFPC/glom and FPW and UPCR, 10 female patients were grouped into 5 age-matched pairs, 3 pairs with identical ages and the 2 other pairs with ages no more than 2 years apart. Except for one pair, in each pair, the subject with lower %NFPC/glom had greater FPW value, suggestive of an inverse relationship between the extent of podocyte injury and the number of NFPC in age-matched Fabry females (Figure 4). Even an 8 year old female with 20% NFPC/glom (case #1) had greater FPW than an 11 year old (case #2) with 50% NFPC/glom. The one exception was a pair with greater FPW in a 14 year old girl with 22% NFPC/glom (case #6) compared to a 16 years old with 13% %NFPC/glom (case #7) (Figure 4). The relationships between %NFPC/glom and UPCR among the above pairs paralleled the relationships between %NFPC/glom and FPW, except for cases #2 and #3 with almost identical %NFPC/glom (50 and 52%, respectively) and slightly (∼15%) greater UPCR for case#2 (Figure 4). However, the relationship between %NFPC and UPCR remained not statistically significant after age adjustment. Also, when adjusted for age, Vv(Inc/FPC) in females correlated with FPW (r = 0.64, p = 0.03). In females with Fabry disease, FPW in electron micrographs where glomerular basement membranes were covered by FPC (562±160 nm) was not statistically different from those where glomerular basement membranes were covered by NFPC (663±283 nm). On the other hand, FPW in either of these areas were greater than FPW in normal control biopsies (406±34 nm, p = 0.037 for FPC and p = 0.048 for NFPC).

## Discussion

We recently demonstrated that the fraction of the volume of podocytes occupied by GL-3 [Vv(Inc/PC)] increases with age in children with Fabry disease and is directly correlated with FPW and to proteinuria,^9^ the latter a strong predictor of renal disease risk among Fabry disease patients.^23^ Increased FPW is a common concomitant of injury in podocytes and is seen in a variety of conditions known to be associated with injury in these cells. [21], [22], [23] Thus careful examination of this cell in Fabry disease is important. This is the first study to provide unbiased electron microscopic morphometric measures of the heterogeneity of podocyte involvement in females with Fabry disease, almost certainly as a result of X-inactivation. Gubler *et al* first described the heterogeneous distribution of GL-3 inclusions in podocytes in 3 females with Fabry disease. [24] Valbuena *et al* also described variable podocyte GL-3 accumulation in 4 females with Fabry disease. [25] However, no quantitative assessment of podocyte GL-3 or statistical analyses was provided in these reports. Similar to these studies, [24], [25] we found that a distinction between Fabry and non-Fabry phenotype, consistent with cells carrying active mutant or wild-type GLA, respectively, could be easily made in podocytes but not in other renal cells of female patients. This could be due to distinct borders of podocyte cell bodies from their neighbor cells, or because podocytes are apparently very long lived [26] and may thus retain GL-3 inclusions for years while other cell types are more frequently replaced.

Importantly, we documented, through unbiased morphometric measurements, that the average fraction of podocyte cytoplasm occupied by GL-3 was virtually identical in male and female patients, suggesting that FPC do not benefit from the enzymatic activity of the adjacent NFPC or from the generally higher residual plasma αGal A in females than on males. [27] [28], [29].

Also important, we found that inter-glomerular variation in podocyte phenotype mosaicism in a given female Fabry patient’s renal biopsy is much smaller than inter-patient variation. This suggests that estimation of podocyte phenotypic mosaicism even in a single glomerulus is representative of this phenomenon in the biopsy and validates the study of podocyte phenotype mosaicism in a few glomeruli. It also suggests that podocyte mosaciasm in females is established relatively early in embryogenesis. It should be noted that in these studies we used random profiles of glomeruli. Although we observed NFPC in patches of up to 5 cells in these two-dimensional glomerular cross-sections, these patches are likely larger in three dimensions. Nonetheless we posit that these patches are not very large, otherwise we might have expected greater inter-glomerular variability as a result of random sectioning through glomeruli.

This study is the first to show a quantitative relationship in females with Fabry disease between the X-inactivation phenomenon and podocyte injury manifest as increased FPW. Although X-inactivation may play a role in the clinical phenotypic expression of X-linked diseases, a direct link between skewed X-inactivation and severity of the Fabry phenotype has been controversial. Dobrovolny *et al*. reported that the trend line between the age and the Mainz severity score index (MSSI) [30] was steeper in 10 Fabry females with *vs*. 28 without unfavorably skewed X-inactivation in leukocytes, urinary and salivary cells. This suggested that random X-inactivation could influence the severity of the Fabry phenotype in female patients. [31] In contrast, Maier *et al*. found that in 46% of the 28 Fabry females studied, skewed X-inactivation did not correlate with phenotype severity. [4] Similarly, in a more recent study, while confirming random X-inactivation in leukocytes in ∼82% of 77 female Fabry patients, there was no relationship between X-inactivation ratios and age, αGal A activity, MSSI scores, cardiac involvement, neuropathic pain or proteinuria. [32] We did not find a statistically significant relationship between % NFPC/glom and UPCR. However, in regression models including UPCR among the predictor variables, UPCR accounted for much less of the variance for rates of GFR decline in women than in men with Fabry disease. [33] Also, increased UPCR can reflect parameters other than podocyte injury, such as impaired tubular reabsorption reabsorption of filtered protein. However, after adjusting for age, we documented a significant inverse relationship between %NFPC/glom and FPW, a widely accepted indicator of podocyte injury. Thus, having more NFPC in glomeruli was associated with less podocyte injury. However, having a greater proportion of NFPC in older females with Fabry disease is not necessarily indicative of less podocyte injury. In fact, the observed increase in the %NFPC/glom with age in female patients in the current study is suggestive of progressive FPC loss with aging due to a survival disadvantage caused by the Fabry phenotype. Thus, glomeruli of older female patients may have fewer total podocytes than those of younger females with lesser %NFPC/glom, a hypothesis that remains to be tested in future studies. On the other hand, our side-by-side comparisons of age-matched female pairs revealed a robust inverse relationship between %NFPC/glom and FPW. However, given that Fabry disease is genetically heterogeneous, with more than registered 600 mutations, [34] it should be expected that attempts to explain all phenotype variation by a single parameter such as mosaicism will necessarily be overambitious. [35], [36] Nevertheless, it will be interesting to examine if a relationship can be found between podocyte mosaicism status in the kidney and skewed X-inactivation in other organs or cell types, especially that of leukocytes. However, some of the cases presented in this study were from historical archived material and no simultaneous blood samples or skin biopsies had been obtained for x-inactivation studies at the time other biopsies were performed.

The relationship between podocyte mosaicism and podocyte injury in female Fabry patients suggests that the status of podocyte X-inactivation, after making adjustment for the age of the patient, could help to identify females with greater risk of progression of Fabry nephropathy. Although it would be desirable to use less invasive methods to obtain information about the proportion of cells with active mutant GLA, the status of mosaicism may be cell type and organ specific. Moreover, as outlined above, studies attempting to link X-inactivation in peripheral blood leukocytes and clinical manifestations of Fabry females have produced contradictory results. [4], [31] Thus far, renal biopsy may be the only reliable way to obtain information about podocyte mosaicism in these patients.

Importantly, the present study showed that not only podocyte mosaicism for Fabry phenotype and podocyte injury are linked in female patients, but also podocyte injury in these patients is not limited to FPC and extends to NFPC, evidenced by increased FPW in GBM areas covered by NFPC compared with normal control biopsies. This is consistent with experimental studies performed by Matsusaka et al. where following administration of the immunotoxin LMB2 to the mice chimeric for hCD25 (LMB2 receptor), not only hCD25+ podocytes, but also hCD25- podocytes were injured and showed foot process widening, suggesting that specific injury to some podocytes can induce non-specific injury to other podocytes, [14] a phenomenon that if severe enough could lead to a vicious cycle of podocyte loss and glomerulosclerosis.

To our knowledge, this is the first report of the finding of rare cells with GL-3 inclusions in kidney biopsies from female Fabry patients. We encountered two females, ages 34 (case #9) and 39 years (case #11), with known Fabry disease by mutation analysis with very rare kidney cells with GL-3 inclusions. One (case #11), had a cardiac variant GLA mutation (N215S) [37] and the other (case #9) had a missense mutation (R112C). Thus, in contrast to males, the finding of even rare cells with GL3 inclusions in kidney biopsies from females should raise strong suspicion for Fabry disease. Notably, both of these subjects had very mild phenotypes, raising suspicion that the favorable skewed X-inactivation (predominantly affecting the mutant copy of GLA) observed in kidney biopsies may have also been present in other organs, especially since R112C mutation has been associated with classical Fabry disease causing ESRD [38], while case #9 in the present study had normal renal function and no history of cardiac disease or strokes. We did not have access to blood samples or other biopsies from these subjects to do correlative studies with our findings in kidney biopsies.

This study has some limitations. Although, it is the largest kidney biopsy study of females with Fabry disease, given the heterogeneity of Fabry mutations and clinical manifestations, additional studies are needed to confirm our results. We did not examine the status of X-inactivation in podocytes directly, rather we studied their apparent phenotype as to presence/absence of GL-3 inclusions in cell body profiles as a surrogate for X-inactivation. However, the easy discrimination between males and females on biopsies based on podocyte mosaicism supports validity of this surrogate. We cannot exclude the possibility of overestimation of %NFPC/glom in this study due to missing GL-3 inclusions in a single section through podocyte cell bodies. However, among 4 biopsies examined from male patients, only 2 of 98 podocyte nucleated profiles, both of very small size, had no GL-3 inclusions. Thus, we believe the extent of %NFPC/glom overestimation due to random sectioning is trivial.

In summary, this is the first study showing that mosaicism of podocytes in females with Fabry disease is related to podocyte injury. The extent of podocyte mosaicism for the Fabry phenotype is quite uniform among the glomeruli. The fraction of the cell body occupied by GL-3 in affected podocytes in females is the same as in males, indicating the absence of significant cross-correction. The relative number of podocytes without the Fabry phenotype increases with age in female patients, suggesting either a disproportionate loss of Fabry-affected podocytes over time and/or selection bias. Information about podocyte mosaicism in kidney biopsies may be useful to identify females with Fabry disease with increased risk of developing progressive podocyte and nephron loss. The methodology we introduced is applicable to future longitudinal biopsy studies to test this hypothesis.
